# ‘Slow down, one detail at the time!’ the influence of reflective-impulsive cognitive style on the recollection of criminal events

**DOI:** 10.1007/s00426-024-02043-7

**Published:** 2024-11-13

**Authors:** Kaja Glomb, Przemysław Piotrowski, Bożena Gulla, Iza Romanowska, Maria Mastek

**Affiliations:** 1 Faculty of Management and Social Communication, Jagiellonian University in Krakow, Krakow, Poland; 2 Social Resilience Lab, Center for Humanities Computing, Aarhus University, Aarhus, Denmark

## Abstract

This study investigates the impact of the reflective-impulsive dimension of cognitive styles on recollection and the accuracy of a criminal events. In a VR-based experimental study, 150 participants were exposed to both criminal and neutral scenarios and subsequently asked to report everything they remembered about them. Cognitive styles were assessed using a computer-administered Matching Familiar Figures Test. The findings highlight that individuals with a reflective cognitive style consistently provided detailed descriptions of the events, irrespective of the context of encoding. In contrast, individuals with an impulsive cognitive style displayed context-specific effects, notably reduced performance when recalling emotionally charged criminal events. Notably, cognitive style did not moderate the accuracy of testimony. Given that the subjects who observed the crime achieved a lower overall accuracy rate than those who observed the neutral video, it appears that accuracy is directly affected by the criminal content.

**Supplementary Information:**

The online version contains supplementary material available at 10.1007/s00426-024-02043-7.

## Introduction

Eyewitnesses play a pivotal role in the criminal justice system, providing critical testimonies that can influence legal outcomes. However, ‘telling the truth and nothing but the truth, poses a challenge, as the act of witnessing a crime is inherently cognitively demanding, requiring individuals to process a multitude of sensory inputs, emotions, and contextual information (Lane, 2006). Eyewitnesses are often thrust into chaotic and emotionally charged situation, where the events unfold rapidly, and their attention is divided among various elements of the scene (Sheleff & Shichor, 1980). The situation often requires the integration of visual, auditory, and sometimes tactile stimuli, the formation of coherent mental representations of the events, and the accurate retrieval of these representations when called upon to testify. Eyewitness testimony is, therefore, a demanding cognitive tasks and a product of complex interplay between perception, diverse memory functions and internal determinants inherent to the individuals themselves (Ghetti et al., 2004). Among them, traits related to perceptual preferences, information processing, and problem-solving – all of which psychologists often categorize under the umbrella of cognitive style.

### Fast and faulty: when cognitive style matters

Cognitive styles refer to individual strategies in problem solving and task performance (Sternberg & Grigorenko, 1997) that people have a proclivity to enter into and which can be adapted to changing environmental demands and can modified based on new experiences (Kozhevnikov, 2007). It encompasses differences in the way information is gathered, processed, organized and used. Depending on the conceptualization of cognitive style, it can be viewed either as a situational predisposition (e.g. Sternberg, 1994) or a relatively stable trait, bearing closer resemblance to the domain of personality (Sternberg & Zhang, 2001). Emphasizing the constancy of cognitive style draws attention to preferences related to both simpler stimulus perception and more demanding, higher cognitive processes, such as categorization and information processing (Zelniker et al., 1976). Although rooted in cognitive functioning, empirical research suggests that cognitive styles remain independent of intelligence (Riding & Pearson, 1994; but also see: Rozencwajg & Corroyer, 2005).

As research on preferences in cognitive styles boasts a rich historical lineage, individuals can be characterized along various categories or dimensions of these styles. Among them is a widely researched spectrum ranging from reflectivity to impulsivity. It highlights the proclivity for processing information at varying speeds and its association with error control (Kagan, 1965; Messer, 1973). Individuals situated at the reflective end of this cognitive style spectrum are characterized by a preference for analytical and meticulous thought processes, displaying a predilection for deliberate and slow information processing while maintaining stringent error control mechanisms (Kagan et al., 1964). Reflective cognitive style is also associated with a more systematic approach to gathering information (Borkowski et al., 1983). In contrast, those within the impulsive style are recognized for their predominantly rapid cognitive processing, favoring a holistic approach to information processing (Kagan et al., 1964; Walczyk & Hall, 1989). While this style can be efficient in some contexts, impulsive individuals may exhibit a higher propensity for errors due to their faster processing speed and potential oversight of finer details. Moreover, considering the resemblance emphasized by Glicksohn and Kinberg (2009) between the impulsive cognitive style and field dependence, it suggests that individuals with this cognitive style might exhibit a heightened susceptibility to salient stimuli in spatial-visual tasks (Quiroga et al., 2011).

While the reflective-impulsive cognitive style has been recognized for its significance in various domains such as learning and academic achievements, workplace performance, and decision-making, limited attention has been directed toward these individual differences within the context of witness testimony. The scarcity of research in this domain represents a noticeable gap in the investigation of what are known as estimator variables—factors that have the potential to influence the accuracy of witness testimony (Wells & Olson, 2003). As cognitive style encompasses inclinations toward both the perception of stimuli and their subsequent processing, it has the potential to influence not only what a witness remember from a criminal event but also their later testimony.

While evidence has established connections between cognitive style and cognitive processes (e.g., Borkowski et al., 1983; Palladino et al., 1997; Pandey & Mishra, 2014) these connections predominantly relate to learning and knowledge acquisition. Consequently, their applicability to eyewitness testimony is uncertain. It is also worth noting that research on eyewitness testimony underscores the importance of analyzing individual differences in cognitive processes, though much of this work has focused on false memories and distortions, as well as susceptibility to suggestion. For instance, Battista et al. (2020) demonstrated that individuals with higher executive functioning, as measured by neuropsychological are more likely to recall accurate details compared to those with lower cognitive resources. Conversely, individuals with diminished executive function are more prone to memory distortions, as they struggle – as the authors suggested – to shift between event details, suppress irrelevant information, and effectively monitor and encode relevant data. Additionally, studies indicate that greater working memory capacity relates to increased resistance to misinformation (e.g. Gerrie & Garry, 2007; Watson et al., 2005). Therefore, given the significance of individual differences in cognitive functioning on witness memory, we believe that exploring predispositions in preferred cognitive styles can further deepen our understanding of the factors that shape the reliability of eyewitness testimony.

Addressing this gap, our study adheres to the recommendation by Yuille and Wells (1991) to align research on eyewitness testimony with real-life scenarios, striving for a contextual resemblance between actual eyewitness experiences and the research environment. To surmount the challenge for ecological validity, inherent to psychology of witness testimony, we have chosen an innovative approach to simulate the realistic context of this experience.

### Matrix: Revolution of eyewitness testimony research

Given the nature of encoding information during witness events, which translates into cognitive, emotional, and motivational demands (e.g. Chae, 2010), a central challenge lies in developing a research method that, while adhering to ethical guidelines, reproduces the complexity of the witness’s experience and maintains control over stimuli and procedural repetitions. Virtual Reality (VR) technology offers a solution by enabling the recreation of crime scenes with a high degree of realism. This affords participants an immersive and more authentic simulation of actual witnessing conditions, as substantiated by its successful application within the broader criminological context (for a review see: van Gelder et al., 2014).

Although, to our knowledge, this technology has not yet been applied in research on cognitive styles, VR has been utilized in memory and learning studies to the effects of cognitive overload (e.g. Barreda-Ángeles et al., 2021; Huang et al., 2020). In the context of witness testimony, this is particularly relevant; as Ihlebæk et al. research (2003) have shown, different memory effects arise when subjects participate in a simulation of a crime compared to when they simply watch a video depicting it. Thus, this suggests the need for a method that enhances the complexity and richness of stimuli, which can be provided by VR (Rizzo & Koenig, 2017).

Keeping its advantages in consideration, we crafted a 360-degree video compatible with immersive VR goggles. This video format significantly broadens the perceptual field, granting participants the ability to survey the entire crime scene from diverse angles, mirroring the complexities of real-world witnessing experiences. Consequently, it offers particular merit for the examination of cognitive style and the underlying information processing mechanisms. The complexity, sensory richness, and dynamic nature of the perceptual field it engenders are likely to evoke heightened participant engagement compared to conventional laboratory research paradigms. Thus, such stimulus manipulation serves to significantly enhance the much-needed ecological validity of the study.

### Current study – variables and predictions

Employing VR technology in crafting a 360-degree immersive video, this study aims to investigate how reflective-impulsive dimension of cognitive style affects recollection of criminal events. Given that the reflective-impulsive cognitive style can influence not only information processing during encoding but also retrieving information from memory when testifying, we incorporated two free recall memory measures into the analyses: (1) the percentage of correctly recalled details relative to the total possible information that could be given, and (2) the accuracy of the testimony. Accuracy rate is defined, after Evans and Fisher (2011) as a number of accurately provided details of the event / Σ accurate + errors. The distinction between correct recollection and accuracy is crucial, as the former focuses solely on the details reported correctly, while the latter evaluates the overall correctness of the testimony by also considering errors in recollection. Therefore, this differentiation holds particular significance in the context of the cognitive style dimension under investigation.

To provide a nuanced understanding of the influence of cognitive styles in eyewitness testimony, we have included a comparison between witnessing a criminal event and a neutral event. We believe this comprehensive approach facilitates a deeper exploration of the interactions between individual differences and situational factors that are believed to elicit emotional responses. In line with Sheleff and Shichor (1980), we posit that even an accidental bystander may become emotionally engaged when witnessing a crime, potentially affecting their cognitive functioning due to heightened arousal (Mather & Sutherland, 2011) or associated unpleasant feelings. Thus, incorporating a neutral condition allows us to assess baseline cognitive processing involved in memory recall without the influence of emotional arousal, providing a clearer understanding of how cognitive styles operate in different contexts. By comparing testimonies for both criminal and neutral events, we can better isolate the effects of emotion on memory performance. Furthermore, this comparison helps determine whether individuals with different cognitive styles exhibit varying levels of memory performance in emotionally charged situations versus neutral ones, given the arguments suggesting a relationship between cognitive styles and emotional reactivity and functioning (see: Zhang, 2008). Indeed, as some studies (e.g., De Jong et al., 1995; Tucker & Newman, 1981) indicate, individuals with a preference for global information processing may exhibit a heightened anxiety sensitivity and psychophysiological response to fear-relevant stimuli. This heightened reactivity to threatening stimuli may consequently impact memory processes by resulting in a diminished memory for secondary details (e.g. Kim et al., 2013; Melcher & Piazza, 2011; for review see: Christianson, 1992).

Our methodology also aligns with the foundational principle of trait activation theory, stating that traits represent latent predispositions that manifest in response to trait-relevant situational cues (Tett et al., 2014). In light of this, one can posit the need to investigate traits across various contexts to explore their potential for activation.

Having this in mind, we formulate predictions regarding the relationship between the variables. Firstly, [H1] we hypothesize an interaction between encoding conditions (Criminal versus Neutral) and Reflectivity that affects the percentage of correctly recalled details. In particular, we expect individuals exhibiting a reflective cognitive style provide more details than those characterized by an impulsive cognitive style, irrespective of the event type [H2]. This hypothesis is grounded in the overall tendency toward analyzing visual stimuli into component details (Zelniker et al., 1977) and systematic processing of information, including sensory input, associated with the reflective cognitive style (Rozencwajg & Corroyer, 2005). Additionally, [H3] we anticipate a decrease the percentage of correctly recalled details in the criminal condition for individuals characterized by an impulsive style. In a context featuring a conspicuous, attention-grabbing stimulus, impulsives with tendency for a global processing may exhibit a bias towards the most salient, threatening stimuli (a crime itself) and this may yield an account of a criminal event that’s less detailed, reflecting a gist-based memory rather than a comprehensive, detail-oriented report. Moreover, as cognitive impulsivity in task performance is often linked to quick reactions without delving into details (Leshem & Altman, 2021), it could encourage brief and concise reports. Furthermore, [H4] we hypothesize that the reflective cognitive style enhances the accuracy of eyewitness testimony, given its bias towards error avoidance at the expense of slower paste of response.

## Materials and methods

### Participants

A total of 153 people participated in the experiment. Due to equipment malfunction 150 participants (f = 95, m = 52, other = 2, prefer not to say = 1; similar female-to-male ratio in both study conditions) were qualified for the final analyses. The study involved young adults (M = 22.1 years old; SD = 2.7). They were given monetary reward for their participation. The participants were unaware of the real purpose of the experiment, and the memory tasks were not mentioned as part of the experimental instructions.

### Materials and apparatus

**Experimental manipulation.** For the study, two videos were prepared: one featuring a criminal scenario and the other a neutral one. This comparison of memories using similar events is inspired by a procedure employed by Houston et al. (2013). The fundamental premise was to create two films illustrating scenarios that primarily differed in the presence or absence of a criminal incident while maintaining consistency in terms of characters and the sequence of actions that lead up to the event. Both scenarios showcased staged events involving the same actors, each lasting approximately three minutes, set in a pub with an outdoor garden.

In the criminal incident video, a male and female “Perpetrators” were involved in a theft from a girl sitting next to them. The male perpetrator engaged the victim by asking for directions while the female perpetrator took a tablet and wallet from the table and left the scene. When the victim discovered the theft and attempted to pursue the female perpetrator, the male stopped her by pushing her onto a chair and knocking the rest of the items off the table. In the neutral condition, the storyline was similar, yet it concluded with a conversation between the main characters (the same actors who played the Perpetrators) and the girl (the same actress who played the Victim), helping them find their way.

Throughout both films, the characters were situated in identical positions, maintaining the same proximity to the witness, adopting similar postures, discussing the same event, performing analogous actions, and making consistent observations about their surroundings. The videos, up to the point of the theft, did not exhibit any significant differences, which were later assessed during a post-event memory performance task. In aiming to elicit negative emotions, the criminal scenario involved the portrayal of the characters as rude and unpleasant. The differences in the depiction of characters primarily related to alterations in their body language—displaying agitated movements and gestures—and modifications in the tone of their comments, rather than introducing additional behaviors or actions.

The videos were shot using a GoPro Fusion camera, which allows to produce high quality 360-degree videos. To present them we used HP Reverbs G1 goggles (head-mounted device) and HP Omen laptop. The video presenting criminal incident was validated in a separate research (Glomb et al., 2023).

**Post-event memory performance** was assessed based on participants testimony given in free recall paradigm. They were asked to recall the scene they previously watched by answering three specific questions: 1)”*What do you remember about the scene in the pub*,* including the events and the people involved?”* 2) “*What do you remember about the appearance of the main characters?“*, 3)”*Is there anything else you remember about the film?“.* The task format was refined based on pilot study results, which indicated that open-ended prompts led to overly brief responses. By specifying three questions, we aimed to encourage more detailed recollections. The second question focused on the perpetrators’ appearance, which is crucial for eyewitness testimony, while the third question provided an opportunity for participants to mention any behaviors of the perpetrators.

Participants’ responses were recorded and transcribed for analysis. The analysis focused on details related to the course of the event, as well as the actions and appearance of the perpetrators. A list of details relevant for scoring was developed in consultation with expert judges, including psychologists not affiliated with the project and a police officer. Each detail was treated as discrete information. Two independent judges—one from within the project and one external—scored the transcriptions by indicating whether the relevant detail was included in the testimony. In cases of discrepancies between the judges, the transcripts were re-evaluated to confirm whether the information was present in the testimonies.

Given the inherent difference in the amount of information presented in the neutral and criminal conditions, a direct comparison of the raw number of recalled details would be misleading. Specifically, participants in the criminal condition were exposed to a greater quantity of details (resulting from more action), potentially inflating raw scores compared to those in the neutral condition. To address this, we normalized the memory performance by calculating the proportion of details recalled relative to the total number of details presented in each condition. This approach allowed for a more accurate comparison of memory performance across conditions by accounting for the differential memory load.

**Kagan’s Matching Familiar Figures Test** (Matczak & Kagan, 1992). The instrument is designed to measure reflection-impulsivity by requiring the subjects to select repeatedly from six alternative figures the one that matches a standard. The number of errors and the time required to complete the test are recorded. We created a reflectivity index based on the Matczak (1982) guidelines for scientific studies on reflectivity-impulsivity. It is derived from the rankings generated by arranging the reaction times observed within the group in ascending order and the number of errors in descending order. The sum of the ranks is an index of reflectivity – the lowest rank implies the lowest reflectivity achieved in the group. The test was administered on 15’’ diagonal computer screen, similarly to research by Hummel-Schluger and Baer (1996).

**Additional measures**. As we simulated criminal event, we wanted to mimic the emotional content of such real-live events. Therefore, we asked subjects to rate the emotions the videos elicited and measured their electrodermal activity during exposure to stimuli as a manipulation check. The results of the between-subjects comparisons are included in Supplement.

### Procedure

The experiment employed a between-subjects design, with participants randomly assigned to either the Criminal or Neutral condition upon enrollment. The procedure consisted of the following steps:

1. *Experimental manipulation*: Participants viewed a video through VR goggles, following a brief preparation session for the VR activity.
2. *Filler task*: Participants completed a self-rating of their emotions and a battery of questionnaires not discussed here.
3. *Post-event memory performance task*, during which participants provided their testimony.
4. *Matching Familiar Figures Test*: Participants completed this task to assess cognitive style.

The time interval between encoding (experimental manipulation) and retrieval (post-event memory performance) was set at 20 min.

The procedure was positively reviewed and approved by the Research Ethics Committee at Institute of Applied Psychology, Jagiellonian University in Kraków before its application (decision number 56/2019 dated 25.11.2019).

The study presented here is part of a broader project that also includes the analysis of additional individual variables (assessed during the Filler task) and alternative memory measures (administered following the MFFT). For a comprehensive overview of all variables and data, please refer to the repository mentioned in the Statements and Declarations section.

## Results

To explore whether individual differences in reflective-impulsive cognitive style influence recollection differently depending on the content of memory, a moderation analysis was conducted. The analysis was preceded by: (1) analysis of individual differences in reflective- impulsive cognitive style; (2) comparison of the percentage of correctly recalled details and accuracy of memory between conditions.

Table 1 presents the absolute measurements of two components comprising a Reflectivity index. The difference between conditions were not significant (Errors: *t*(148) = − 0.461; *p* = .645; Reaction time: *t*(148) = 0.090; *p* = .929).

**Table 1:** The scores of two components of reflectivity index obtained by the subjects (*N* = 150) in both study conditions

Component		Mean	SD
Response time	Criminal	17.990	7.754
	Neutral	17.881	7.099
Errors	Criminal	1.240	2.078
	Neutral	1.387	1.808

*Note* The *response time* was calculated as the median response time for a figure

Then, we compared the percentage of correctly recalled details in the Criminal and Neutral conditions. There was a significant difference between the conditions (*t*(148) = -3.595, *p* < .001, *d* = − 0.587), with participants who observed the neutral event recalling relatively more details (M = 0.23; SD = 0.079) than those who witnessed the criminal incident (M = 0.18; SD = 0.069).

**Fig. 1:**
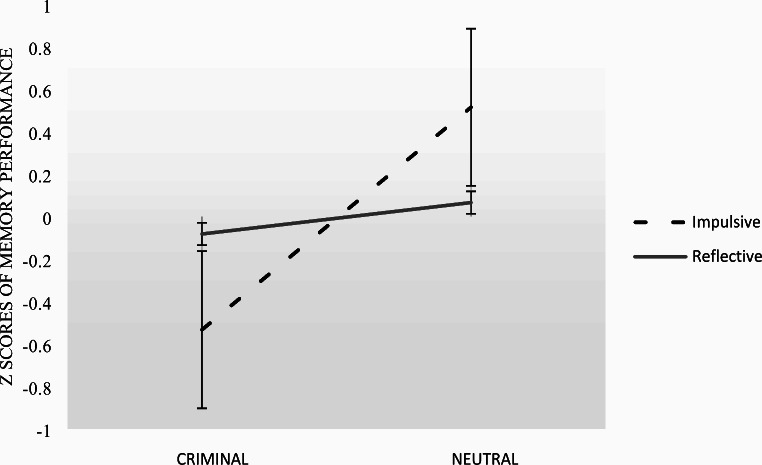
Interaction plot depicting the effect of reflective-impulsive cognitive style on relationship between condition and the percentage of correctly recalled details. *Note* The plot was made using standardized data

Next, we conducted the moderation analysis. We included the following variables in the model: research condition (IV), the percentage of correctly recalled details about the event (DV), reflectivity (Moderator). The model summary revealed an overall significance of the model (R^2^ = 0.116, *F*(3,146) = 6.412, *p* < .001) and no effect of reflectivity on memory performance (coeff = 0.000, *p* = 0.995). Moreover, the analysis showed a significant interaction between cognitive style and condition (*coeff* = 0.000, *t*(146) = -2.442, *p* = .016), indicating the role of reflectiveness. To better understand the interaction, we examined conditional effects and interaction plot (Fig. 1).

When Reflective index is low (participants who are categorized as ‘impulsives’, the condition has a strong effect on recollection (effect = 0.081, *se* = 0.019, *p* < .001). In contrast, in the case of individuals scoring high in reflectivity, the effect of the experimental condition on recollection is reduced and insignificant (effect = 0.011, *se* = 0.018, *p* = .521). This relation between variables is presented in Fig. 1. In essence, individuals with a reflective cognitive style performed at a consistent level of success during memory task, regardless of condition. On the other hand, individuals with impulsive cognitive style exhibited diminished memory performance in the case of an criminal event. There is also a noticeable difference in variation between high and low scorers, with reflective individuals showing reduced variation in memory performance.

We repeated these analysis for accuracy as well. Although we found significant difference between study conditions (M_Criminal_ = 0.85, SD_Criminal_ = 0.09; M_Neutral_ = 0.94; SD_Neutral_ = 0.07; *t*(148) = -6.692, *p* < .001, *d* = -1.09); the interaction between moderator and condition did not prove to be significant (*coeff* = 0.000, *se* = 0.000, *p* = .748). This suggests that the accuracy of recollection remains under the direct effect of the type of experience recalled.

Additionally, to investigate the potential direct influence of the reflective-cognitive style on the overall number of errors in testimony, we conducted correlation analyses. These analyses revealed no significant relationship between reflectivity index and the total number of errors in testimony in the general (*r* = .126, *p* = .126) and in the individual study conditions (Criminal: *r* = .186, *p* = .110; Neutral: *r* = .050, *p* = .672). This suggests that, contrary to our hypothesis, a more reflective cognitive style does not appear to significantly reduce the number of errors in recall, regardless of the context in which the information is processed.

## Discussion

In this study, we examined how individual differences in reflective-impulsive cognitive style affect two measures of memory recallection. We specifically focused on how this relationship varies depending on the context of memory encoding—criminal versus neutral events. Our findings indicate that this cognitive dimension significantly influences the percentage of correctly recalled details, with its impact on memory performance varying based on the type of event witnessed. Thus, we consider our first, main hypothesis to be supported. To the best of our knowledge, this dimension has not been studied in witness testimony psychology using a similar approach. Therefore, we reference existing literature while acknowledging the need to consider its relevance to eyewitness testimony carefully.

Our results demonstrate that individuals characterized by a reflective cognitive style—marked by deliberate and systematic information processing—perform consistently in memory tasks, regardless of the type of event. Supporting this finding is the absence of a condition effect on the percentage of correctly recalled details and the relatively low variance in results across conditions, especially when compared to individuals with an impulsive cognitive style. Therefore, this results suggest that reflective style may promote the ability to encode and retrieve many details, even in emotionally charged or chaotic situations, such as witnessing a crime.

In contrast, context-specific effects were observed for individuals with an impulsive cognitive style, which supports our third hypothesis. This style is primarily defined by a higher speed of information processing, global thinking, as well as a propensity for making mistakes. Our study showed that when the event involved criminal (and thus emotional) content, the proportion of correctly reported details by individuals with this style was significantly lower than when the event was neutral.

This finding indicates that the reflective style contributes to stability in memory performance, further reinforcing the notion of enhanced meta-memory competence among individuals exhibiting this cognitive style (Borkowski et al., 1983). However, as we did not observe reflectives superiority in memory performance in neutral condition, our hypothesis is therefore only partially confirmed. Our study may therefore cautiously suggest that reflective style is a protective factor against the negative impact of emotions on cognitive processes. It is possible that by isolating details in perceptual field, a characteristic of analytical processing, they are less affected by fear-related, salient stimulus, thus they are able to provide a more accurate and detailed-oriented report. However, it is worth noting that research on the analytical-holistic dimension suggests that the analytical style seems to promote better performance on cognitive tasks, although success requires the availability of sufficient processing capacity (Riding et al., 2003; Viator et al., 2020). Thus, results observed among young people in a cognitive prime may not fully capture the relationship between the variables in general.

As demonstrated by interaction plot, we also revealed that while in neutral condition impulsives present higher level of memory performance than reflectives (contrary to our Hypothesis 2), they are sensitive to the emotional salience of the event, as the percentage of correctly recalled details was significantly influenced by criminal event. Our findings, thus, indirectly supports the previous studies on fear-related sensitivity of impulsives (e.g., De Jong et al., 1995) and observed negative association between analytical processing and dispositional fear (Mayiwar et al., 2023). Furthermore, considering the possible alignment between impulsive and field-dependent cognitive styles, the findings may cautiously be associated with Karp’s (1963) classical research, suggesting a subtle yet discernible correlation between factors related to the resistance of distraction and the capacity to overcome embeddedness—a cognitive mechanism characterizing field independence. It is plausible that in an emotional context, individuals with impulsive tendencies are more susceptible to distractions, thereby potentially impeding the accurate recall of a greater quantity of details. However, among individuals characterized by this cognitive style, we did not observe a tendency towards more errors in the criminal condition. This suggests that during free recollection and without time pressure, the testimony of those with impulsive tendencies seems not only to produce fewer both – accurate details and errors.

Our study highlights the importance of considering the emotional context when investigating memory. As we have shown that cognitive styles may interact with emotional experiences, impacting eyewitness testimony, the results could have significant implications for the legal system. It suggests that not all eyewitnesses are equally affected by the emotional intensity of a crime. Thus, understanding the interplay between cognitive styles and emotional contexts can aid in assessing the credibility of eyewitness testimony and, subsequently, making more informed legal decisions. We, therefore, believe that this research could reignite the discourse on the significance of individual differences in witness testimony, which, particularly regarding fixed traits, often yields mixed and occasionally contradictory results. It is our view that this might be attributed to the prevailing research methodology, frequently reliant on correlational studies or regression analyses while overlooking the inclusion of a control condition. We believe that our proposed methodology offers a greater opportunity to detect situation-specific effects of individual differences on eyewitness testimony.

However, this study did not reveal the significance of reflective style in error control mechanisms. Therefore, the fourth hypothesis is not supported by the data. The accuracy of testimony was influenced by the type of event rather than by cognitive style. This indicates that errors in recollection may be driven primarily by the emotional or situational cues present in the content of the memory rather than by the individual’s cognitive style. Consequently, further research is needed to investigate how individual differences may affect errors in testimony.

### Limitations and Future Research

We believe that this research present robust evidence of the impact of cognitive styles on memory function, yet recognizing the study’s constraints remains essential. We conducted a single experiment primarily focused on reaction time and performance errors related to reflective-impulsive measurements. Further research should explore alternative operationalizations of cognitive style and its potential influence on memory in diverse contexts.

Another limitation of this study is the inherent difference in complexity between the neutral and criminal conditions. While the inclusion of a neutral condition facilitates the investigation of the interaction between cognitive styles and encoding context, it is important to acknowledge that criminal events typically involve more details and higher emotional arousal. This disparity could influence participants’ attention and memory encoding processes, potentially confounding the results. However, we believe that our analytical approach mitigates these concerns. By focusing on the normalized number of recalled details and considering the proportion of correctly and incorrectly recalled information, we aimed to protect against drawing unsupported conclusions.

Moreover, the sample used in this study consisted of young adults. This demographic does not fully represent the diversity of individuals who serve as eyewitnesses in real-world legal cases, thus future research should aim to include a broader range of age groups and backgrounds to ensure the generalizability of the findings.

Furthermore, since we did not find a significant interaction between the type of event and reflectivity on memory accuracy, further research is warranted. Future studies could involve manipulating the testimony scenario to examine the impact of errors. There remains the possibility that the reflective cognitive style might serve as a protective factor when errors lead to adverse outcomes for individuals involved.

Despite these limitations, we believe that our study presents a case supporting the claim that the impulsivity-reflectivity dimension of cognitive style, with its underlying information processing preferences, is among the potential factors influencing eyewitness testimony.

## Electronic supplementary material

Below is the link to the electronic supplementary material.

Supplementary Material 1

## Data Availability

The data (DOI 10.17605/OSF.IO/3PHVF) supporting this research is available on the Open Science Framework website under a CC-By Attribution 4.0 International license. Link to the data: https://osf.io/3phvf/.
