# Supplementary material for: ‘Slow down, one detail at the time!’ the influence of reflective-impulsive cognitive style on the recollection of criminal events
Source: Psychol Res. 2024 Nov 13;89(1):6. doi: 10.1007/s00426-024-02043-7 (PMC11557669; doi:10.1007/s00426-024-02043-7)
Supplement: Supplementary file 1 — Supplementary Material 1 [file 426_2024_2043_MOESM1_ESM.docx]

Supplement

To confirm the effectiveness of our experiment in terms of evoking emotions, we administered two manipulation checks reflecting two component of emotional reaction: arousal and subjective feeling.

**Arousal.** In this study, electrodermal activity (EDA) was measured. We used a wireless Shimmer3 GSR+ unit (worn as a wristband on the non-dominant hand) and two EDA diodes attached to the middle and index fingers. The unit was calibrated with a maximum sampling rate frequency of 1092 Hz. To reduce noise interfering with the measurements, the subjects were asked to take a comfortable position, place their forearms on the desk, and attempt to minimize hand movement while watching the video.

EDA analysis began with an initial review of the data for any measurement irregularities that may have been caused by equipment dysfunction or incorrect diode insertion. As a result, two records (one per condition) were eliminated.

Considering the procedure we applied, namely exposure to the film, which was designed to elicit general arousal not necessarily related to a specific, single stimulus, we analysed the Skin Conductance Response (SCR) based on the frequency of peaks recorded in fixed time-periods (Braithwaite et al., 2013). In order to eliminate inter-individual differences in electrodermal activity, we also decided to compare the measurements recorded when subjects were watching the Crime/Neutral video with the measurement collected at the beginning of the study, when subjects watched a neutral, relaxing film (baseline). We chose two segments lasting 165 seconds for the comparison. We did not consider the first several seconds of the videos in order to eliminate the initial arousal associated with the novelty effect of the stimulus.

The extraction of tonic and phasic EDA components was performed based on the methodology proposed by Hossein Aqajari et al. (2021). The data were then checked for possible abnormal readings and outliers suggesting interference during data collection, significant movements and other possible artifacts. As a result, two records per condition were eliminated. Thus, 144 records (72 per condition) were included in the final analysis.

Table S1 presents the results of the electrodermal analysis. To compare the arousal between-subjects, we conducted independent t-tests for two samples. First, we compared the absolute number of amplitudes recorded in subjects while watching the film (*t*(142) = 1.713; *p* = .088, *d* = .286). Second, we compared the difference in the number of peaks between the film and the baseline (*t*(142) = 2.669; *p* = .008, *d* = .445). The results indicate that the change in arousal between the first and second measurements was significantly higher for the Crime condition. Further supporting this claim is the filtered measurement of electrodermal activity presented in Figure S1, which indicates higher skin conductance values in this condition. A similar course of arousal, with higher amplitudes at certain points in the film, reflects discrete differences in the presentation of the characters, who in the crime condition were expected to make an impression as more unpleasant that in neutral condition.

**Table S1**

*Results of electrodermal activity analysis (N = 144)*

|  |  | Crime  n = 72 | | Neutral  n = 72 | |
| --- | --- | --- | --- | --- | --- |
|  |  | *M* (*SD*) | *Min - Max* | *M* (*SD*) | *Min - Max* |
| Number of peaks | Baseline | 13.36 (1.60) | 9 – 17 | 13.75 (1.12) | 11-17 |
|  | Film | 13.51 (1.54) | 10 – 16 | 13.10 (1.38) | 10-16 |
| Difference | | .15 (2.04) | -4 – 6 | -.65 (1.55) | -5 – 2 |

**Figure S1**


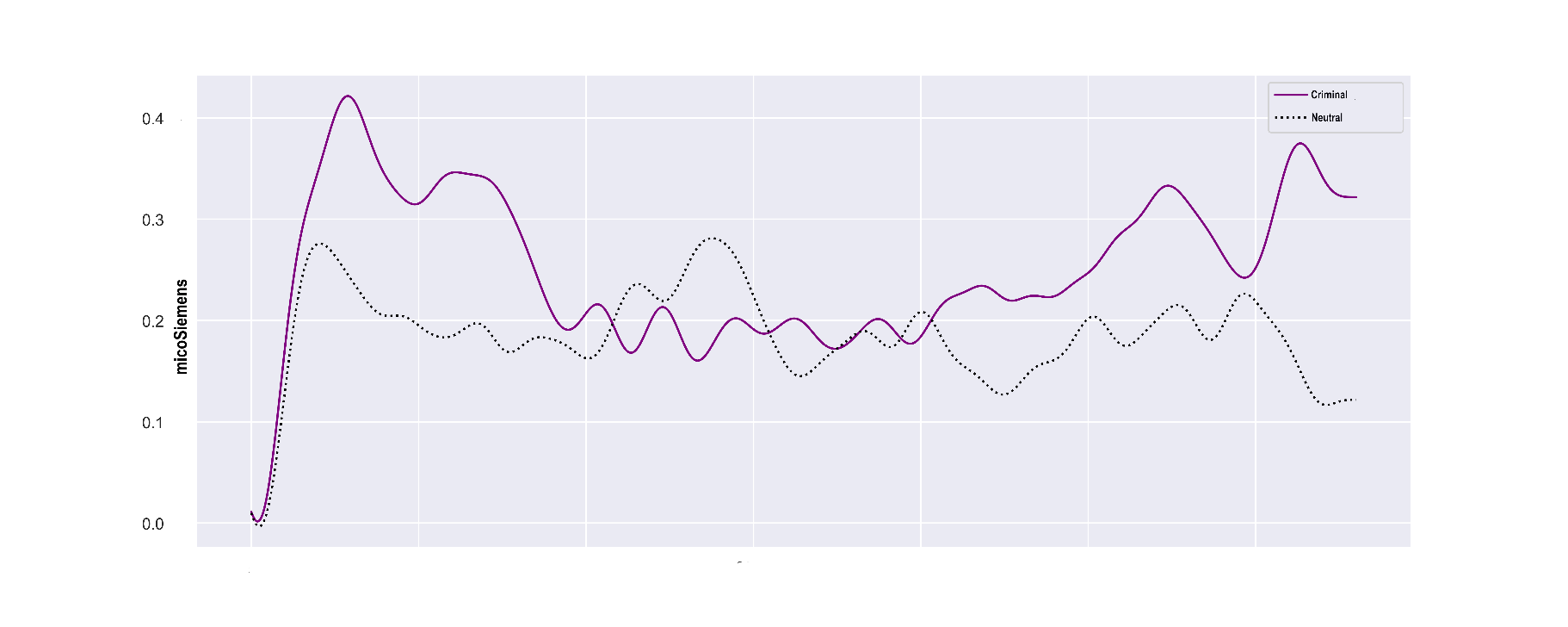
*Filtered phasic activity recorded while watching the video. Between-subjects comparison*

**Subjective feeling**. We used the Geneva Emotion Wheel (Sacharin et al., 2012) to determine the valence and intensity of emotions experienced by respondents while watching the film. It is a self-report measure consisting of discrete emotion labels corresponding to emotion families that are arranged in a circle divisible into four quadrants. The alignment of emotion terms is fundamental to the two-dimensional space limited by the axis of valence (negative to positive) and control (low to high). As a result, the method allows us to measure four domains of emotions (negative/low control, negative/high control, positive/low control, and positive/high control). The response options correspond to different levels of intensity for each emotion family, from low intensity (1) to high intensity (5). Subjects can also indicate that they did not feel a particular emotion (0) and they can independently label the name of the emotion they experienced. The final scores of these four domains are calculated as the mean rating values obtained for all discrete emotions that comprise these domains.

Immediately after watching the video, participants were asked to identify discrete emotions and rate the intensity they felt while watching the video. To answer the question about the difference in emotional response between witnessing a crime scene and a neutral scene, we created a negative emotion (NE) index, which is the average score of all 10 discrete emotions available on the GEW with negative valence (Anger, Hate, Contempt, Disgust, Fear, Disappointment, Shame, Regret, Guilt, and Sadness). Additionally, we counted two additional indexes that differentiate negative emotions on the dominance dimension, which allows more detailed conclusions about the relationship between the type of crime and witness reactions. As a result, we were able to compare the low-control (Disappointment, Shame, Regret, Guilt, and Sadness) and high-control (Anger, Hate, Contempt, Disgust, and Fear) negative emotions between subjects. We also counted the positive emotions (PE) index as an additional manipulation check This is the average intensity of all the positive emotions experienced while watching the film.

To compare the emotional reactions of witnesses to a crime and those observing a neutral scene, we performed Student's t-tests for independent samples. The results are presented in Table S2. Participants who observed the crime event felt stronger negative emotions – in general and both types of negative emotions in terms of dominance (high and low) – than those who watched the similar neutral event. Moreover, the results suggest no difference in positive emotions ratings between subjects.

**Table S2**

*The average results of the emotion intensity ratings in each study condition and the between-subjects comparison (N = 150)*

| Emotion | Crime  M (SD) | Neutral  M (SD) | Between subjects comparisons  t(148) |
| --- | --- | --- | --- |
| NE | 1.22 (.91) | .22 (.37) | 8.84*; *p* < .001, *d* = 1.44 |
| NE high | 1.46 (1.07) | .24 (.51) | 8.90*; *p* < .001, *d* = 1.45 |
| NE low | .99 (1.05) | .20 (.35) | 6.15*; *p* < .001, *d* = 1.03 |
| PE | .93 (.69) | .97 (.80) | .35; *p* = .727 |

Notes: * due to the violation of equal variation assumption a Welsh t-test with Satterthwaite approximation for the degrees of freedom was used.

NE – Negative Emotion index; NE high – Negative Emotion with high control index; NE low - Negative Emotion with low control. PE – Positive Emotion index.

Reference

Braithwaite, J. J., Watson, D. G., Jones, R., & Rowe, M. (2013). A Guide for Analysing Electrodermal Activity (EDA) & Skin Conductance Responses (SCRs) for Psychological Experiments. Psychophysiology, 49(1), 1017–1034.

Hossein Aqajari, S. A., Naeini, E. K., Mehrabadi, M. A., Labbaf, S., Dutt, N., & Rahmani, A. M. (2021). pyEDA: An Open-Source Python Toolkit for Pre-processing and Feature Extraction of Electrodermal Activity. Procedia Computer Science, 184, 99–106. https://doi.org/10.1016/j.procs.2021.03.021

Sacharin, V., Schlegel, K., & Scherer, K. R. (2012). Geneva Emotion Wheel Rating Study. https://archive-ouverte.unige.ch/unige:97849
